# Do Europeans consider sustainability when making food choices? A survey of Polish city-dwellers

**DOI:** 10.1017/S1368980019000326

**Published:** 2019-03-12

**Authors:** Krystyna Rejman, Joanna Kaczorowska, Ewa Halicka, Wacław Laskowski

**Affiliations:** Department of Organisation and Consumption Economics, Faculty of Human Nutrition and Consumer Sciences, Warsaw University of Life Sciences (WULS-SGGW), Nowoursynowska 159 C Street, 02-776 Warsaw, Poland

**Keywords:** Sustainable diet, Consumer, Food choice determinants, Poland

## Abstract

**Objective:**

To obtain a better insight into the conceptualization of sustainable consumption among consumers with special focus on food choice determinants. Previous studies show that people present positive attitude towards sustainable diet while their everyday food choices do not follow sustainable diet rules.

**Design:**

A structured questionnaire was designed and data were collected via computer-assisted telephone interview among a random group (*n* 600) of city-dwellers. Quota sampling was used in proportion to the city population. Cluster analysis (*k*-means method) was applied to identify consumer segments according to the determinants of food choices. Consumer segments were identified using one-way ANOVA with *post hoc* Duncan comparison of mean scores and cross-tabulation with *χ* ^2^. The Friedman test was applied to identify enablers and barriers of sustainable food choices.

**Setting:**

Cities in Mazovia, the best developed, central region of Poland.

**Participants:**

Adults (21–70 years old).

**Results:**

Consumers are not familiar with the concept of sustainability and are not able to define it adequately. Only 6 % of the studied population indicated that sustainable consumption is connected with nutrition which has possibly less impact on the environment. Three segments of consumers were distinguished regarding their attitude to food choice determinants adequate to sustainable diet: Non-Adopters (17 %), Emergents (32 %), Adopters (51 %). Desire to improve health by decreasing body weight was the main driver for sustainable food choices, while prices were the main limitation.

**Conclusions:**

Knowledge dissemination on sustainability issues is needed to empower consumers to make more sustainable food choices and to make public health and food policy measures more effective.

‘Sustainable development’, as described for the first time in *The Limits to Growth* report commissioned by the Club of Rome thinktank and published almost 50 years ago^(^ ^1^ ^)^, is linked to economic growth, natural environment imbalances and climate change due to excessive emission of greenhouse gases. The widely cited conception of sustainable development was defined in the 1987 report *Our Common Future*, summarizing discussions of the World Commission on Environment and Development, chaired by Gro Harlem Brundtland. It highlighted three fundamental components to sustainable development: environmental protection, economic growth and social equity. In the report sustainable development was defined as ‘a development that meets the needs of the present without compromising the ability of future generations to meet their own needs’^(^ ^2^ ^)^. However, a great deal of interest in sustainability issues has been expressed globally in recent years^(^ ^3^ ^)^.

There is no doubt that part of the responsibility for the status of the environment rests with people in relation to the consumption of many categories of goods. Food and drink consumption has been recognized as an environmentally significant behaviour^(^ ^4^ ^)^, taking into account all activities undertaken along the food supply chain. It is commonly believed that dietary choices can make an important contribution to meeting current environmental challenges^(^ ^5^ ^)^. Especially dietary patterns and consumer behaviour in developed countries result in excessive use of water and greenhouse gas emissions, soil degradation and food wastage. According to estimates of the EIPRO (Environmental Impact of Products) project, food and drink consumption accounts for 20–30 % of the ecological footprint of individuals in the EU^(^ ^6^ ^)^ and to the same extent in other developed countries^(^ ^7^ ^)^. Meat and meat products (including poultry, sausages, etc.) have the most adverse effect on the environment out of all food groups consumed, followed by milk and dairy^(^ ^6^ ^)^. So a healthier diet, with lower meat and dairy consumption, might have less environmental impacts^(^ ^8^ ^)^. The ‘classical’ triple focus on economic (profit), social (people) and environmental (planet) goals does not quite fit the complexity that is raised by the challenge of sustainability. Health is missing or not emphasized sufficiently, which is also true for important cultural dynamics such as quality, taste and social life^(^ ^9^ ^)^. In the early 1980s at the University of Giessen the concept of ‘wholesome nutrition’ was developed, that included four equally important aspects: health, ecology, economy and society. The concept referred to ‘a mainly plant-based diet, where minimally processed foods are preferred. The central food groups are vegetables and fruits, whole-grain products, potatoes, legumes and dairy products. Native cold-drawn plant oils, nuts, oleaginous seeds and fruits are also important, but should be consumed in moderate quantities. If desired, small amounts of animal-based foods (meat, fish and eggs) can be consumed’^(^ ^10^ ^)^. The concept evolved towards ‘sustainable nutrition’ and took into account the fifth dimension of culture because the respective cultural background influences food habits^(^ ^10^ ^)^. When the term ‘sustainable diet’ was first used, it referred to diets which are healthy for both the environment and people^(^ ^11^ ^)^. The current definition of sustainable diet, proposed by the FAO and Biodiversity International, is more complex and refers to diets which ‘are protective and respectful of biodiversity and ecosystems, culturally acceptable, accessible, economically fair and affordable; nutritionally adequate, safe and healthy, while optimising natural and human resources’^(^ ^12^ ^)^. Implementing sustainable diet principles will allow to meet the challenges arising from public health nutrition and environmental sustainability issues. Inadequate dietary behaviours contribute to 1·4 billion overweight and obese people in the world^(^ ^13^ ^)^ and at the same time contribute to food insecurity. A large body of nutritional epidemiological evidence associates a Western dietary pattern with the rise of diet-related non-communicable diseases^(^ ^14^ ^)^.

Sustainable food choices give an opportunity to combine sustainability messages with public health messages^(^ ^15^ ^)^. They come down to several rules that are largely consistent with dietary guidelines for the population. While dietary guidelines remain primarily health focused, synergies between health and sustainability mean that the guidelines do include implicit sustainability messaging^(^ ^16^ ^)^. In addition to tips regarding healthy food choices – which are included in the dietary guidelines in many countries or developed by non-governmental organizations (NGO), for example:

∙ Eat less meat/Moderate your meat consumption, both red and white, and enjoy other sources of proteins such as peas, beans and nuts;
∙ Eat more plants/Prefer plant-based foods;
∙ Eat less processed food/Eat fewer foods high in fat, salt and sugar;
∙ Eat a variety of foods

– the rules directly focus on environmental or social aspects of sustainability, for example:

∙ Eat better food (Sustain; a British NGO)/Buy food that meets a credible certified standard; consider MSC, free-range and fair trade (WWF)/Organic foods and fair trade products (Germany)/Value your food, ask where it comes from and how it is produced (UK)/Choose fish sourced from sustainable stocks, seasonality and capture methods are important here too (UK);
∙ Seasonal and regional – your first choice (Germany)/Buy local, seasonal and environmentally friendly food (Sustain)/Urban, rural, regional – sustainable farming with short transport routes and seasonal products (Germany);
∙ Drink tap water (UK)/Tap water is the drink with the lowest environmental impact (Sweden)/Tap water is drinking-water (Germany);
∙ Waste less food (WWF)/Aim to be waste-free: reducing food waste and packaging (Sustain)/Don’t waste food (UK)^(^ ^10^ ^,^ ^17^ ^–^ ^22^ ^)^.

The current national food-based dietary guidelines in Poland – presented as the Pyramid of Healthy Eating and Physical Activity together with ten tips for healthy eating and updated in 2018^(^ ^23^ ^)^ – do not consider sustainable or environmental issues and focus on the prevention of chronic CVD, obesity, diabetes and cancer. Among these guidelines, some can be considered as corresponding to sustainable consumption: ‘Eat fruits and vegetables as often as possible and as much as possible, at least half of what you eat and remember the right proportions: 3/4 – vegetables and 1/4 – fruits’; ‘Eat cereal products, especially whole grains’; ‘Drink at least two large cups of milk every day (you can substitute them with yoghurt, kefir and – partly – with cheese)’; ‘Limit your meat consumption (especially red and processed meat products to 0·5 kg per week), choose fish, pulses and eggs’; ‘Avoid sugar and sweets (replace them with fruit and nuts)’. The base of the Pyramid is physical activity and vegetables and fruits are the most recommended food group. However, many country-wide (such as the Federation of Polish Food Banks) and regional NGO undertake activities aimed at reducing food waste, promoting local and seasonal food, and encouraging consumers to drink tap water.

Consumers play a major role in making the food chain more sustainable through appropriate food choices and many of them perceive sustainability as a positive and valuable concept^(^ ^24^ ^–^ ^27^ ^)^. Nevertheless, studies show that there is a gap between consumers’ positive attitude towards this concept and market behaviour, and most important, everyday consumption practices are likely to be resistant to change^(^ ^24^ ^,^ ^28^ ^,^ ^29^ ^)^. In emerging market economies, like in Poland, dynamic changes in the food market, evolving consumption patterns and social inequalities in the last decades make this issue an even greater challenge. The Polish food and drink industry has undergone many deeply restructuring processes in the last 30 years, such as privatization, concentration and globalization. Since the early 1990s global agri-food companies started to play a crucial role in introducing research innovations, new technologies as well as marketing, management and organization solutions in the sector. The development of the food market was accompanied by changes in the food consumption pattern, which are typical for countries in the period of dynamic economic growth. The changes consisted mainly of increases in the consumption of meat, fats and highly processed foods. In the period 2000–2017, according to the Polish Central Statistical Office data^(^ ^30^ ^)^, the consumption of high-protein animal products (meat, fish, milk and eggs) increased by 12·3 % (in weight by 34·3 kg per capita), while the consumption of cereals, potatoes, fruits and vegetables decreased by 16·5 % (in weight by 70·6 kg per capita). These changes are exactly opposite to the sustainable food consumption guidelines.

With this in mind, the present study aimed to obtain better insight into the conceptualization of sustainable consumption by Polish consumers with special focus on food choice determinants relevant to the concept of sustainability.

## Materials and methods

### Measures

For data collecting a structured questionnaire consisting of three parts was constructed. The questionnaire was piloted on a convenience sample of ten people and the corrections were made where ambiguities occurred. The first part of the questionnaire dealt with understanding of the concept of sustainable food consumption. The respondents were presented with four options (definitions) out of which three were not correct. In the correct answer (option C), i.e. ‘everyday diet is carried out so as to minimize the influence on the natural environment’, the definition of sustainable food consumption had been deliberately simplified and narrowed down to the environmental dimension, since its fulfilment is crucial to achieve economic and social benefits. The inclusion of all complex aspects of sustainability into the proposed options could be too suggestive and draw the respondents’ attention straight to this answer.

The second part of questionnaire focused on different categories of food choice determinants. The third part involved respondent features, including gender, age, education level, type of employment, size of the city, average monthly income and self-evaluation of household financial condition.

### Sample and fieldwork

Respondents were selected from adults aged 21–70 years living in big cities with more than 50 000 inhabitants, bearing in mind that: (i) the concept of sustainable consumption is known so far to just a small part of society; (ii) the population of city-dwellers usually represents higher level of education, achieves higher income, etc. and therefore is more open to new ideas; and (iii) new consumer trends, also in the field of food and nutrition, spread from the inhabitants of large cities to the rural population, for which they constitute a certain model of future food consumption and behaviour. It was further decided that the area of research implementation should be one region, to avoid differences in the level of socio-economic development of the cities as well as the standard of living of their residents. Finally, the Mazovia region, the best developed region in the country (in terms of gross domestic product and growth, unemployment rate, personal income and education level), was chosen as the research area. The region is centrally located in Poland and includes seven big cities with a population exceeding 50 000 inhabitants: four cities with over 50 000 to 100 000 people; two cities with over 100 000 to 500 000 people; and Warsaw, the capital and the largest city in the country, with about 1·7 million inhabitants. The quota sampling method according to the cities’ population was used and in the case of Warsaw according to the number of inhabitants in eighteen city districts. The sample consisted of 600 adult city-dwellers and Warsaw inhabitants constituted 75 % of the sample. Data were collected using the computer-assisted telephone interview method in 2014. Obtaining the sample required performing over 15 800 telephone calls, and half of them ended with a refusal. On average, the time of one interview took 18min. Sociodemographic details of the study participants are shown in Table 1.

**Table 1:** Sociodemographic and economic characteristics (%) of the total sample and clusters identified in the adult (21–70 years old) city-dwellers (*n* 600) from Mazovia, central Poland, 2014

	Total sample (*n* 600)	Non-Adopters (*n* 100)	Emergents (*n* 196)	Adopters (*n* 304)	*P* value	*χ* ^2^	df
Gender					0·000	36·04	2
Male	38·3	61·0	42·3	28·3			
Female	61·7	39·0	57·7	71·7			
Age					0·000	54·771	6
18–35 years	15·8	35·0	19·4	7·2			
35–50 years	25·3	26·0	27·0	24·0			
50–65 years	42·2	33·0	36·7	48·7			
>65 years	16·7	6·0	16·8	20·1			
Education					0·004	11·293	2
Middle and high school	41·2	27·0	40·8	46·1			
University degree	58·8	73·0	59·2	53·9			
Income (monthly)					0·004	25·761	10
<1250 PLN*	20·4	12·0	15·0	26·6			
1251–2000 PLN	34·4	28·3	38·5	33·8			
2001–3000 PLN	16·2	17·4	15·5	16·2			
>3001 PLN	29·0	42·4	31·0	23·4			
Place of living (population size)					0·003	16·412	4
City, <100 000	10·5	6·0	13·8	9·9			
City, >100 000–500 000	14·8	7·0	11·7	19·4			
City, >500 000	74·7	87·0	74·5	70·7			
Occupation							
Own-account work	14·2	18·0	16·3	11·5			
White-collar	43·8	60·0	45·4	37·5			
Blue-collar or unemployed	10·8	9·0	10·2	11·8			
Retired	31·2	13·0	28·1	39·1			

* 1000 PLN=approximately US$ 270 or €250.

### Statistical analyses

Data were analysed using the statistical software package IBM SPSS Statistics version 21.0. Cluster analysis (*k*-means method) was applied to identify consumer segments according to the determinants of their food choices. Consumer segments were identified using one-way ANOVA with *post hoc* Duncan comparison of mean scores and cross-tabulation with *χ* ^2^. The Friedman test was applied to identify enablers and barriers of sustainable food choices.

## Results

### Factors influencing food choices

**Fig. 1:**
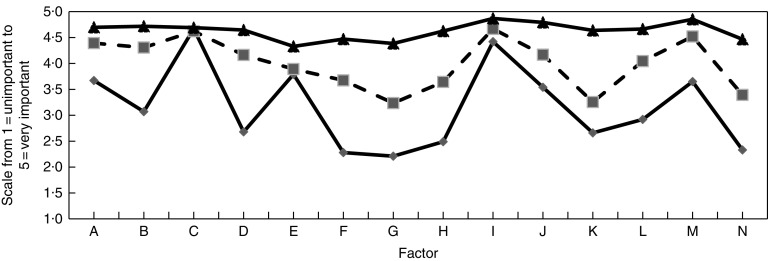
Mean evaluation scores of factors influencing food choice in Non-Adopters (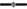), Emergents (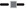) and Adopters (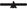). In 2014, adult (21–70 years old) city-dwellers (*n* 600) from Mazovia, central Poland were asked: ‘How important are the following factors influencing food choice? Please assess the influence of each factor using the scale: very important (5), quite important (4), cannot say (3), quite unimportant (2), unimportant (1)’. Factors: A=food safety; B = protection of natural environment*; C=taste; D=methods of food cultivation, animal breeding, food processing; E=price; F=organic production; G=place of catching fish; H=returnable or recyclable packaging; I=food quality; J=origin of food to support domestic/Polish producers; K=origin of food to limit transport; L=ethical standards in production, processing and selling food; M=health/healthy nutrition; N=quality assurance certificates on food packaging. *Underlining indicates factors that significantly differentiate respondents in designated clusters

A 5-point Likert scale was used to analyse the role that fourteen factors played in the respondents’ food choices. Eight of them referred to sustainable behaviours (Fig. 1).

The most important factors influencing food choice of interviewed consumers were taste and food quality (99 % of respondents indicated that both were ‘important’ or ‘quite important’). Healthy nutrition, food safety, origin of food products to support local/Polish producers and natural environment protection were indicated by more than 90 % of participants and scored above 3·0. Slightly fewer respondents reported that they paid attention to price (88 %), the way the purchased food was grown/bred/processed (85 %) and to the maintenance of ethical standards in the food chain (84 %). Less important factors, indicated by 70 % of respondents, were reusable packaging, organic method of production, buying local/Polish products to limit food transport and quality assurance certificates. The least important factor influencing the food choices of study participants was the place where the purchased fish is caught (65 %).

### Cluster analysis

Cluster analysis was used to segment study participants according to food choice determinants. The analysis revealed no statistical difference (*P*=0·209) for one variable only, taste, that turned out to be a very important factor (mean score in subsequent clusters=4·67, 4·62 and 4·69, respectively). Differences for the remaining thirteen factors were statistically significant (on the level of *P*<0·001) and these led to the identification of three consumer segments. Mean evaluation scores for each cluster are shown in Fig. 1. Based on the importance of sustainability-related issues in their food choices, the segments were labelled Adopters (51 % of participants), Emergents (32 %) and Non-Adopters (17 %).

The three identified clusters showed significant differences with respect to gender, age, education and income level and employment (Table 1). In the Adopters cluster, in which food choices were based on the impact on own health and the natural environment in the highest degree, 72 % were women as compared with 58 % in Emergents and 39 % in Non-Adopters (*P*=0·000). People aged ≥50 years constituted 69 % of the Adopters cluster, 54 % of the Emergents and only 39 % of the Non-Adopters (*P*=0·000).

As shown in Fig. 1, in the case of Adopters the three top-scoring factors influencing food choice were: food quality (mean=4·87), healthy nutrition (mean=4·85) and buying local food to support domestic producers (mean=4·80). Only four factors were evaluated as ‘quite important’ with mean scores close to the border value of 4·5 (price, mean=4·33; place of catching fish, mean=4·38; possession of quality assurance certificate, mean=4·46; and organic production, mean=4·47).

Respondents in the Adopters’ segment expected the purchased food products not only to be of good quality, have a beneficial influence on health and taste good, but also to be produced with low environmental impact. Adopters also wanted food to be produced in a way that respects biodiversity and ecosystems as well as ethical standards in the food supply chain.

The Emergents cluster included people whose food choices are driven by healthy nutrition, environmental protection and the method of purchased food cultivation/animal breeding/food processing. However, these consumers do not consider the full impact of their purchasing behaviour on natural environment: one-third of them reported that buying local/domestic products to limit food transport is ‘unimportant’ or ‘quite unimportant’ when choosing food. The three determinants mentioned above (and only these ones) were reported by Emergents as moderately important when choosing food (mean score=3·26, 3·23 and 3·39, respectively). About one out of four (23 %) of these participants described purchasing products in reusable packaging as ‘unimportant’ or ‘quite unimportant’, which resulted in the entire Emergents segment reporting this factor as being quite important (mean=3·64).

The third of the identified clusters, Non-Adopters, downplayed the importance of sustainability in their food choices and diet-related behaviour. This is the only group where the mean evaluation score of choice factors was below 3. Low scores were given to seven of the eight detailed determinants that characterize a sustainable diet. Only in the case of a general statement ‘protection of environment’ was the mean score slightly higher (3·07). The key determinants of food choice for the Non-Adopters (apart from taste) were product quality and price: 96 and 78 %, respectively, answered either ‘very important’ or ‘quite important’.

### Barriers to sustainable food consumption choices

In order to identify the barriers to sustainable food choices, respondents were asked to rank factors (on a 5-point scale, with the end points 1=‘least important’ and 5=‘most important’) that would stop them from introducing changes to their current diet. Responses were analysed using the Friedman test, with statistically significant level of *P*=0·01 (Table 2). Among the entire sample, the factors were divided into groups in which there was no statistically significant difference between statements. The three strongest barriers (group A) were excessively high food prices, belief that their diet is good/healthy and habit. Weaker barriers (group B) in the respondents’ view comprised low availability of recommended food products; and the weakest barrier (group C and D) was lack of knowledge on how diets can be changed.

**Table 2:** Barriers* to sustainable food choices according to cluster and in the total sample of adult (21–70 years old) city-dwellers (*n* 600) from Mazovia, central Poland, 2014

	Non-Adopters (*n* 100)	Emergents (*n* 196)	Adopters (*n* 304)	Total sample (*n* 600)
Excessively high prices	A	A	A	A
Belief about having good/healthy diet	AB	A	AB	A
Force of habit	A	A	BC	A
Poor assortment and low availability	BC	B	C	B
Lack of knowledge on how diet can be changed	C	C	D	C

* There is no statistically significant difference among statements marked with the same letter (Friedman test): A is the most important statement; D is the least important statement.

The key barrier to change, for all identified clusters, was high prices of the recommended foods. Habits constituted an equally important barrier for Non-Adopters and Emergents. Additionally, Emergents believed that their diet is good/healthy. The least important barrier in all three segments was insufficient knowledge on how to change diet.

In this context it should be noted that only 35 % of the study participants reported understanding the term ‘sustainable diet’. To explain the term, respondents were asked to choose one out of the following four suggested response options:

1. energy value of daily food consumption equals energy expenditure (option A);
2. the share of vegetable products and animal products in the diet is equal (option B);
3. everyday diet is carried out so as to minimize the influence on the natural environment (option C); and
4. the cost of food is adjusted to financial capabilities (option D).

Among those respondents who reported to know the term, 43 % chose the wrong answer (i.e. the definition of an energy-balanced diet, option A) and only 18 % (i.e. 6 % of the whole sample) understood it in the correct way (option C). It is worth mentioning that the correct answer concerning understanding of the term sustainable diet presented to the respondents had been simplified on purpose. Only the impact of diet on the natural environment was taken into account, because typical consumers link food with nature and agriculture. They do not identify the economic and social implications of sustainable diets such as food security or the well-being of next generations.

### Drivers of sustainable food consumption choices

Respondents were also asked to rank statements that would encourage them to change their current diet and make it more sustainable. Statistical analysis of the results (Friedman test, *P*=0·01) is presented in Table 3. The most encouraging factors (group A) were: the need to improve health, lower prices and knowledge on the need to change diet into a more plant-based one. Acquiring information on food products produced in ethical/sustainable way was less encouraging (group B). The least encouraging factors (group C and D) turned out to be social campaigns advocating actions to protect the environment.

**Table 3:** Drivers* of sustainable food choices according to cluster and in the total sample of adult (21–70 years old) city-dwellers (*n* 600) from Mazovia, central Poland, 2014

	Non-Adopters (*n* 100)	Emergents (*n* 196)	Adopters (*n* 304)	Total sample (*n* 600)
Need to improve health, including decreasing body mass	A	A	A	A
Lower prices	B	A	A	A
Knowledge on the need to make diet more plant-based	C	A	B	A
Getting information on food products produced in an ethical/sustainable way	C	B	C	B
Social campaigns encouraging to undertake actions aimed at protecting the environment	C	C	D	C

* There is no statistically significant difference among statements marked with the same letter (Friedman test): A is the most important statement; D is the least important statement.

Adopters reported that the most encouraging factors that would convince them to make more sustainable food choices were the need to improve their health and lower prices of food products. Emergents indicated that apart from lower prices and the need to improve health, they would be encouraged by widespread knowledge on the need to make their diets more plant-based. In the Non-Adopters’ view the need to improve health (including losing weight) was the key factor driving changes in their current food consumption. People in all three consumer segments reported that social campaigns had a limited effect on consumption change.

## Discussion

The present study brings new insights on factors that influence food choices associated with sustainable food consumption, barriers to and encouragers of change in Polish city-dwellers. These insights can help design comprehensive programmes to develop sustainable food consumption in Poland and other countries, although further research is needed to identify cultural differences.

Most of the interviewed Polish city-dwellers (65 %) were not familiar with the term sustainable diet and 72 % of those who had heard of it understood it in the wrong way: 43 % answered that it is an ‘energy-balanced diet’. This observation reveals a general lack of knowledge about sustainability and the impact of food choices on health and the environment. Low awareness of consumers in this respect was also pointed out in other studies^(^ ^31^ ^–^ ^33^ ^)^, which showed that a sizeable part of European consumers find it hard to define the term ‘organic food’ in an adequate way. Comparative studies conducted on a sample of consumers from six EU countries (UK, France, Germany, Spain, Sweden and Poland) also demonstrated a limited understanding of the concept of sustainability^(^ ^34^ ^)^.

The outcome of empirical studies has revealed that while making their food choices, consumers primarily take into consideration taste, quality, impact on health and safety. More altruistic drivers such as environmental protection or support for local producers tend to play a secondary role. Studies showed that environmental awareness in Poland is on a low level; for example, the need to limit food waste in households is seen from the perspective of own/individual budget rather than the ecological^(^ ^35^ ^,^ ^36^ ^)^. There is a widespread belief that routine frequent shopping behaviours result in a rational (down-to-earth) approach to food purchases (i.e. having in mind own benefits) with social and ethical issues becoming less important. Comparative national studies in seven European countries (Netherlands, UK, Sweden, Ireland, Belgium, Latvia, Germany) confirm that taste and quality are the key drivers of food choice followed by health issues (six countries) and environmental issues (five countries)^(^ ^37^ ^)^.

Niva *et al*.^(^ ^38^ ^)^ note that a number of factors simultaneously shape the decision of food choice. Consumers can choose a diet that partially or entirely follows the rules of sustainable consumption indirectly for other reasons. They can buy seasonal or local produce because of their better taste; choose organic products for health-related benefits^(^ ^39^ ^)^; or reduce meat consumption because of empathy for animals^(^ ^40^ ^)^ or its high price. Such behaviour reveals the complex character of daily choices of food and diet that are made not according to clearly defined priorities, but as a result of a compromise between a number of different values, possibilities and requirements.

Our analysis of a large survey of the determinants of food choice among adults revealed differences in Polish city-dwellers’ concern over how their food purchasing behaviour impacts the natural environment. Exploring the differences led us to identify three food consumer segments: Non-Adopters, Emergents and Adopters. Differences in consumer beliefs about their consumption-related behaviour and its impact on the natural environment, and how it might affect food safety and the good health of present and future generations, have been described in earlier publications^(^ ^38^ ^,^ ^41^ ^–^ ^43^ ^)^. Such attitudes have been called ‘perceived consumer effectiveness’. In the present study of Polish city-dwellers, consumers who are acutely aware of the impact of their food choice decisions are more willing to engage in sustainable growth initiatives. This group of people more often take into consideration the ecological, economic and social aspects of obtaining and producing foods they buy.

The present study suggests that Adopters consider sustainability issues (natural environment and their own health) to a larger degree while choosing foods and pay more attention to how human health and ecosystems interact. The majority of Adopters were middle-aged women (50–65 years old), which is in line with the characteristics of sustainable food consumers described by other researchers^(^ ^4^ ^,^ ^39^ ^,^ ^42^ ^,^ ^44^ ^–^ ^47^ ^)^. According to Niva *et al.* ^(^ ^38^ ^)^ this consumer group is most sensitive about the issues related to food and health, which is visible in the ‘activities of sustainable food consumption such as eating seasonal fruit and vegetables, buying organic products, reducing meat consumption, etc.’. On the other hand, it can be assumed that this group (compared with younger consumers) is also more willing to resign from hyperconsumption or to ‘show off’ (e.g. buying exotic food or products with excessive packaging). The present study results, similar to those of Niva *et al.* ^(^ ^38^ ^)^, did not confirm the impact of education and income on sustainable food choice, although the works of Haanpää^(^ ^44^ ^)^, Onyango *et al.* ^(^ ^45^ ^)^ and Wier *et al.* ^(^ ^39^ ^)^ demonstrate the presence of such correlation. Food prices can be an obstacle in implementing a sustainable diet as an increasing discrepancy between the costs of healthy and non-healthy foods is observed^(^ ^48^ ^)^. More costly diets are of higher healthy quality, in both developed- and middle-income countries^(^ ^49^ ^)^. In high-income countries and in emerging economies over the last 30 years, the cost of healthy items has risen more than that of less healthy options, thereby encouraging diets that lead to excess weight^(^ ^50^ ^)^. Meta-analysis covering studies in ten countries also provided evidence that healthier foods and consistently healthy diets cost more than less healthy options^(^ ^51^ ^)^.

Previous studies have shown that everyday consumption practices are likely to be resistant to change^(^ ^28^ ^)^. Consumer willingness often does not translate into sustainable consumer behaviour because of a variety of barriers have been identified, including availability, affordability, convenience, product performance, conflicting priorities, scepticism and force of habit^(^ ^42^ ^,^ ^48^ ^)^. Grunert^(^ ^24^ ^)^ analysed possible barriers to sustainable food choices and among them discussed the lack of awareness and/or credibility and lack of motivation at the time of making food choice decisions. It seems that motivation is the biggest bottleneck in making healthy food choices and the major factor explaining discrepancies between attitude and behaviour^(^ ^34^ ^)^. Studies also reveal a strong connection between ‘familiarity’ and liking of foods as a barrier to sustainable food choices^(^ ^52^ ^)^. Our studies show that the level of consideration for sustainability is lowest in the Non-Adopters cluster (constituting 17 % of the sample), consisting mostly of men, and should be the target for change. In our research the most important barriers to change towards a more sustainable food consumption model were linked to consumers’ beliefs that prices of recommended products are too high and that their own diet is ‘good and healthy enough’. Respondents indicated that if they needed to decrease their body mass and if prices of recommended food products were lower, they would be motivated to change their choices. Other studies have also shown that price is among the main barriers to purchase and consume sustainable products which are carbon- or eco-labelled^(^ ^24^ ^,^ ^53^ ^)^. So, in public health nutrition programmes the promotion of sustainable diet should focus on each aspect as it is stated in the FAO’s definition, among them accessibility, affordability and cultural acceptance. According to Mertens *et al.* ^(^ ^54^ ^)^, when designing an optimized sustainable diet both facets of nutritional health should be taken into account: the essential nutrients that are consumed and the important acceptable foods for maintaining nutrient intake and promoting health.

The importance of integrating environmental considerations into dietary guidelines for populations in different countries is now recognized as an important component of a policy response concerned with health, food security and environmental sustainability^(^ ^55^ ^)^.

## Conclusions

In recent years, the growing concern for environmental issues and food insecurity has emphasized the need to promote sustainable diets. Following the sustainable diet rules in everyday diet is also an opportunity to reduce the occurrence of diet-related diseases. Taking into consideration all these dimensions sustainability has become a priority issue for public health nutrition^(^ ^13^ ^)^. Our research found significant differences among adult Polish city-dwellers regarding their consideration for sustainability in their diets. In general, familiarity with the recommendations of sustainable food consumption is low (only 6 % of respondents correctly defined the term) and the most important determinant of food choice is taste. However, the presented cluster analysis identified a group of consumers (Adopters, constituting 51 % of the sample) which was more aware of the impact of food purchased on their own health and the natural environment and took these factors into consideration when making purchasing decisions. This group consisted mostly of women and consumers aged ≥50 years.

According to the latter, sustainable food choices and consumption require knowledge and financial means; hence the social and economic disparities within society may be an important barrier to spreading the idea of sustainable consumption. From the public health perspective, the concept, goals and challenges of sustainable consumption are closely linked to preventing diet-related diseases and improving the health of both adults and children. More research in the field of sustainable diet in EU countries and the cooperation of governmental, public health-oriented institutions and NGO in the development of a comprehensive EU system approach to raise awareness and promote change towards sustainable food choices are needed. A better understanding of the importance of sustainable concerns in consumer food choices can transfer to beneficial effect on dietary patterns and health status of the population.
